# Supplementary material for: Acute Effects of Brevetoxin-3 Administered via Oral Gavage to Mice
Source: Mar Drugs. 2023 Dec 16;21(12):644. doi: 10.3390/md21120644 (PMC10744354; doi:10.3390/md21120644)
Supplement: Supplementary file 1 [file marinedrugs-21-00644-s001.zip › marinedrugs-2711672-Supplementary Figures S1-S4.pdf]

marine drugs

*Supplementary Materials*

# Acute Effects of Brevetoxin-3 Administered by Oral Gavage to Mice

**Peggy Barbe [1], Jordi Molgó [1], Robert Thai [1], Apolline Urman [1], Denis Servent [1], Nathalie Arnich [2] and Mathilde Keck [1,*]**

[1] Université Paris Saclay, CEA, INRAE, Département Médicaments et Technologies pour la Santé (DMTS), SIMoS, 91191 Gif-sur-Yvette, France; peggy.barbe@cea.fr (P.B.); jordi.molgo@cea.fr (J.M.); robert.thai@cea.fr (R.T.); apolline.urman@cea.fr (A.U.); denis.servent@cea.fr (D.S.); mathilde.keck@cea.fr (M.K)
[2] Risk Assessment Directorate, ANSES (French Agency for Food, Environmental and Occupational Health and Safety), 94701 Maisons-Alfort, France; nathalie.arnich@Anses.FR
* Correspondence: mathilde.keck@cea.fr, Tel.: +33 1 69 08 99 62

## Supplementary Figures

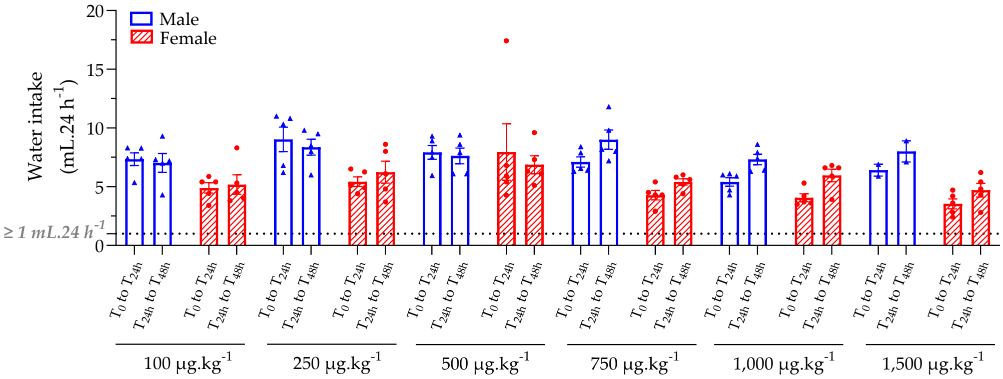


**Figure S1:** Swiss mouse water intake per 24 h after BTX-3 oral administration for each group (BTX doses are indicated, female are represented in red striped histograms) over 48 h. Mixed-effects model followed by Dunnett's multiple comparisons test was used; non-significant effects observed; $n$ = 5 mice/group, except for male at 1,500 µg.kg$^{-1}$ where 3 mice were euthanized at $T_{4h}$.

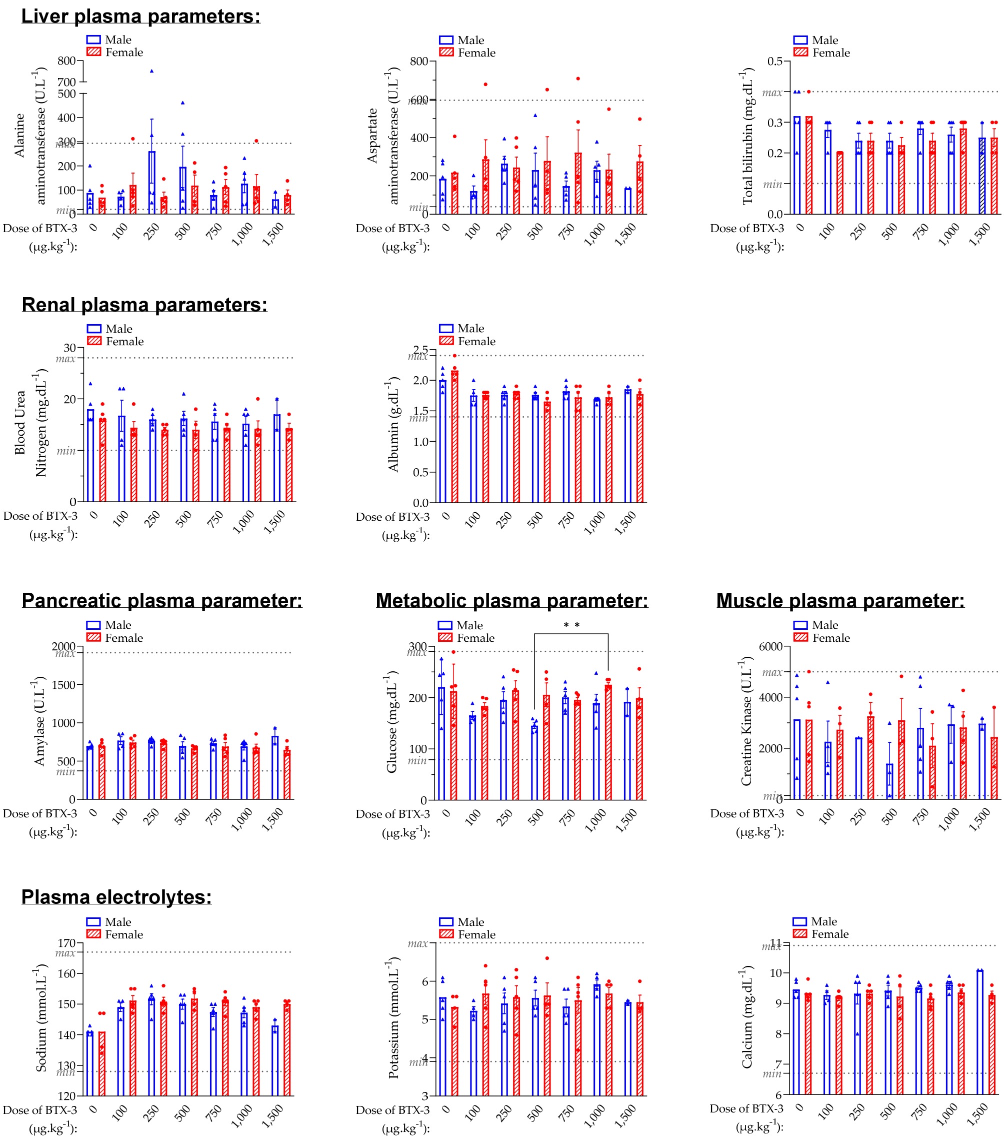


**Figure S2**: Clinical chemistry in the Swiss mouse analyzed 48 h after BTX-3 administration in each group studied (doses are indicated, female are represented in red striped histograms). The dashed lines (in grey) indicate thresholds (min and max) for the parameters examined. The Kruskal-Wallis test followed by Dunn's multiple comparisons test was used; ** $P < 0.01$; $n$ = 4-5 mice/group, except for male at 1,500 µg.kg$^{-1}$ where 3 mice were euthanized at $T_{4h}$.

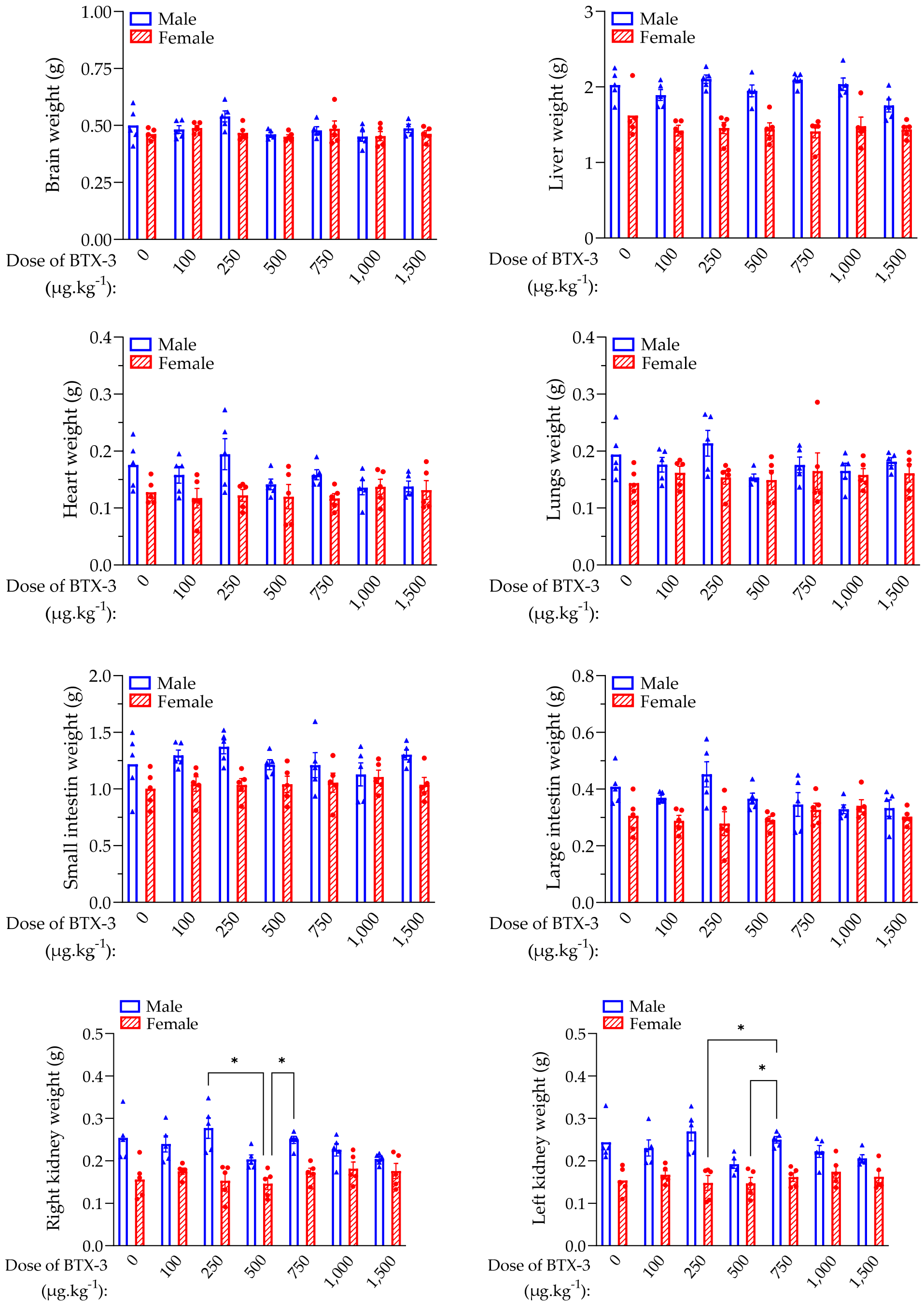


**Figure S3**: Mouse organs weight measured 48 h after BTX-3 administration in each group (BTX-3 doses are indicated, female are represented in red striped histograms). The organs of the three male administered with 1,500 μg.kg$^{-1}$ were measured when euthanized (4 h after administration). The Kruskal-Wallis test followed by Dunn's multiple comparisons test was used; * $P < 0.05$~~, ** $P < 0.01$~~; *n* = 5 mice/group.

**(a)**

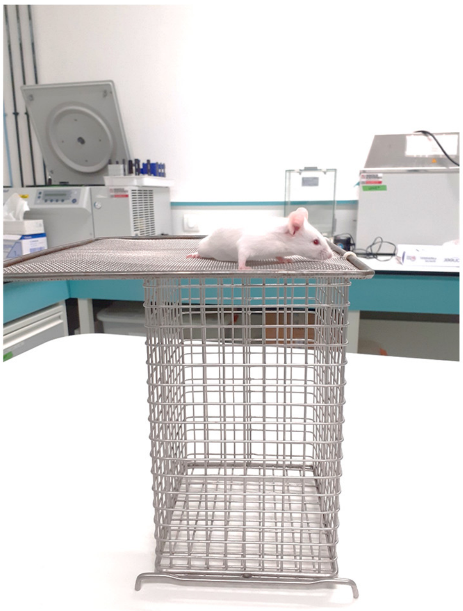

**(b)**

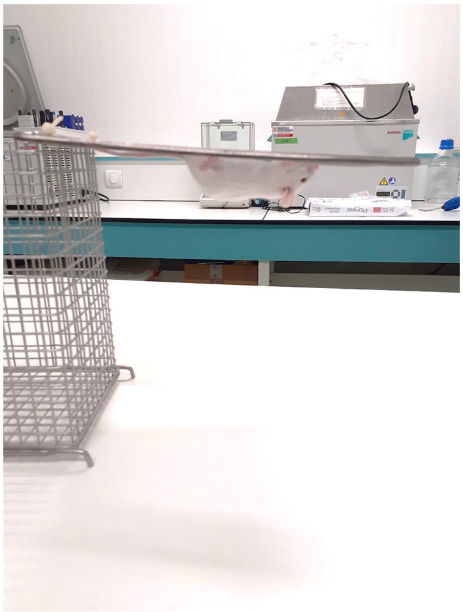

**Figure S4**: The grip test equipment used in the study. Mouse was placed on the grid **(a)**, and then the grid was turned over **(b)** to observe success or failure with attempted collision or failure without attempted collision under the 5 sec after the grid was turned around.
