# Supplementary material for: Acute Effects of Brevetoxin-3 Administered via Oral Gavage to Mice
Source: Mar Drugs. 2023 Dec 16;21(12):644. doi: 10.3390/md21120644 (PMC10744354; doi:10.3390/md21120644)
Supplement: Supplementary file 1 [file marinedrugs-21-00644-s001.zip › marinedrugs-2711672_Report S1_BMDL10 BTX-3 muscle activity for 2 sex.pdf]

Report

Analysis name: BTX-3 muscle activity 2 sexes
This report was generated by **Anonymous** on **8/7/2023 3:52:54 PM (CET)**. PROAST version 70.1

Input values

**Removed data**	No
**Type of response data**	Quantal
**Dose column(s)**	dose
**Response column(s)**	Incidence
**Group size column(s)**	N
**Covariate column**	Sex
**Litter effect**	No
**BMR (CES)**	0.1
**Model averaging**	Yes
**Number of bootstrap runs**	200
**AIC criterion**	2

Graphical output

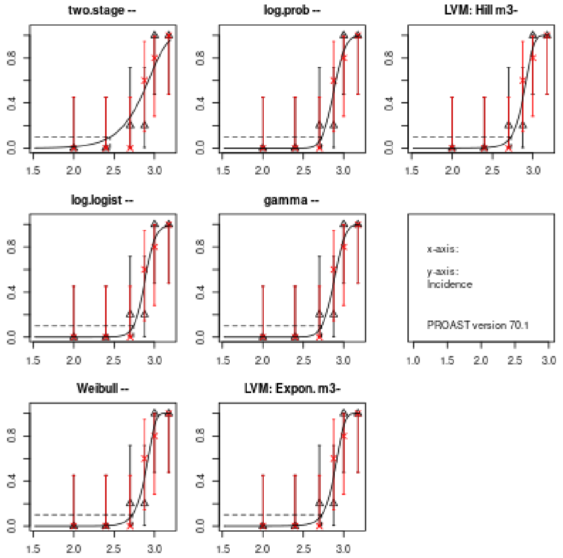

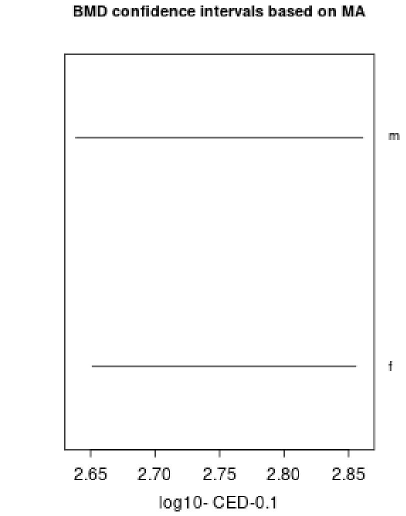

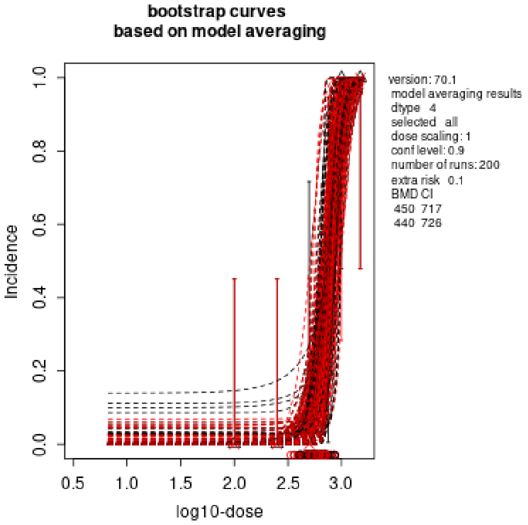

Fitted models

model	No.par	loglik	AIC	accepted	BMDL	BMDU	BMD	sens.subgr	conv
null	1	-40.38	82.76	NA	NA	NA	NA	NA	NA
full	12	-14.02	52.04	NA	NA	NA	NA	NA	NA
two.stage	3	-16.88	39.76	no	NA	NA	284	--	yes
log.logist	3	-13.67	33.34	yes	411	702	546	--	yes
Weibull	3	-13.32	32.64	yes	374	646	528	--	yes
log.prob	3	-13.65	33.3	yes	407	704	533	--	yes

model	No.par	loglik	AIC	accepted	BMDL	BMDU	BMD	sens.subgr	conv
gamma	3	-13.51	33.02	yes	394	694	531	--	yes
LVM: Expon. m3-	3	-13.36	32.72	yes	379	665	534	f	yes
LVM: Hill m3-	3	-13.37	32.74	yes	379	666	534	f	yes

Model weights

model	weight
two.stage	0.0055
log.logist	0.1358
Weibull	0.1926
log.prob	0.1385
gamma	0.1593
EXP	0.1851
HILL	0.1832

BMD confidence interval based on model averaging

Sex	BMDL	BMDU
f	448	717
m	435	726
